# Establishment of a human pluripotent stem cell-derived MKX-td Tomato reporter system

**DOI:** 10.1186/s13287-022-03203-5

**Published:** 2022-11-12

**Authors:** Yuki Fujisawa, Lu Ming, Daisuke Yamada, Tomoka Takao, Takeshi Takarada

**Affiliations:** Department of Regenerative Science, Okayama University Graduate School of Medicine, Dentistry and Pharmaceutical Sciences, Okayama, 700-8558 Japan

**Keywords:** Tendon, Human pluripotent stem cells, Tenocytes, Tendon regeneration

## Abstract

Tendon regeneration is difficult because detailed knowledge about tendon progenitor cells (TPCs), which produce tenocytes to repair tendon tissue, has not been revealed. Mohawk homeobox (MKX) is a marker of TPCs or tenocytes, but a human pluripotent stem cell (hPSC)-based reporter system that visualizes MKX^+^ cells has not been developed. Here, we established an hPSC-derived MKX-tdTomato reporter cell line and tested the induction ratio of MKX-tdTomato^+^ cells using our stepwise/xeno-free differentiation protocol. MKX-tdTomato^+^ cells were generated with high efficiency and expressed tendon-specific markers, including *MKX, SCX, TNMD,* and *COL1A1*. Our MKX-tdTomato hPSC line would be a useful tool for studying the development or regeneration of tendon tissue.

**Supplementary Information:**

The online version contains supplementary material available at 10.1186/s13287-022-03203-5.

## Introduction

Tendons are fibrous connective structures composed of collagen fibers that connect muscles to bones. Tendons are easily damaged by injury, overuse, or age-related degeneration, and tendinopathy is common and hard to recover from due to the poor regenerative potential of tendons [1, 2]. An effective treatment to induce tendon regeneration is still needed to improve patients’ quality of life. In recent decades, cellular therapies have been proposed as a promising approach to overcome tendon defects [3]; cell types commonly used in tendon healing include mesenchymal stem cells, tendon stem/progenitor cells, induced pluripotent stem cells (iPSCs), and embryonic stem cells (ESCs) [4–8]. Although several tenogenic differentiation protocols using pluripotent stem cells (PSCs/ESCs) have been reported, none of them describe the expansion capacity of these differentiated cells.

During embryogenesis, paraxial mesoderm is considered to differentiate into several cell types, including skeletal muscle cells, chondrocytes, osteocytes, dermal fibroblasts, and tenocytes [9]. Tenocytes are specific fibroblast cells that constitute the tendon. Some researchers have recently reported the successful differentiation of pluripotent stem cells (PSCs)-derived tenocytes in vitro [7, 10] and their promising therapeutic applications, indicating important insights into tendon regeneration. In this study, we used an induction protocol for the efficient differentiation of the paraxial mesoderm into tenocytes derived from human pluripotent stem cells (hPSCs).

Mohawk homeobox (MKX) is an essential transcription factor that is persistently expressed during tendon development and plays a crucial role in tendon maturation and maintenance [11, 12]. Here, we established an MKX-tdTomato reporter hPSC line to generate tenocytes using our tenogenic differentiation protocol.

## Materials and methods

### Cell culture

Human ESC cell line SEES4 (donated by RIKEN BRC. Japan) was cultured and maintained using StemFit (AK02N, Ajinomoto). Before reaching subconfluency, the cells were dissociated with TrypLE Select (Thermo Fisher)/0.25 mM EDTA and suspended in StemFit containing 10 µM Y-27632. The cells (1 × 10^4^) were then suspended in StemFit containing 10 µM Y27632 and 8 µl iMatrix511 (human laminin-511 E8 fragment, Nippi) and added to a 6 cm dish. Next day, the culture media were replaced with fresh StemFit without Y-27632. After that, the media were replaced every two days until the next passage (Table 1).

**Table 1:** Oligos for PX459-MKX gRNA

Purpose	Name	Sequence
MKX 3'UTR knock-in	hMKX-CRPs2	caccGGCTAATAAGCATATGGCGT
	hMKX-CRPa2	aaacACGCCATATGCTTATTAGCC

### Establishment of an MKX-tdTomato reporter cell line

**Fig. 1:**
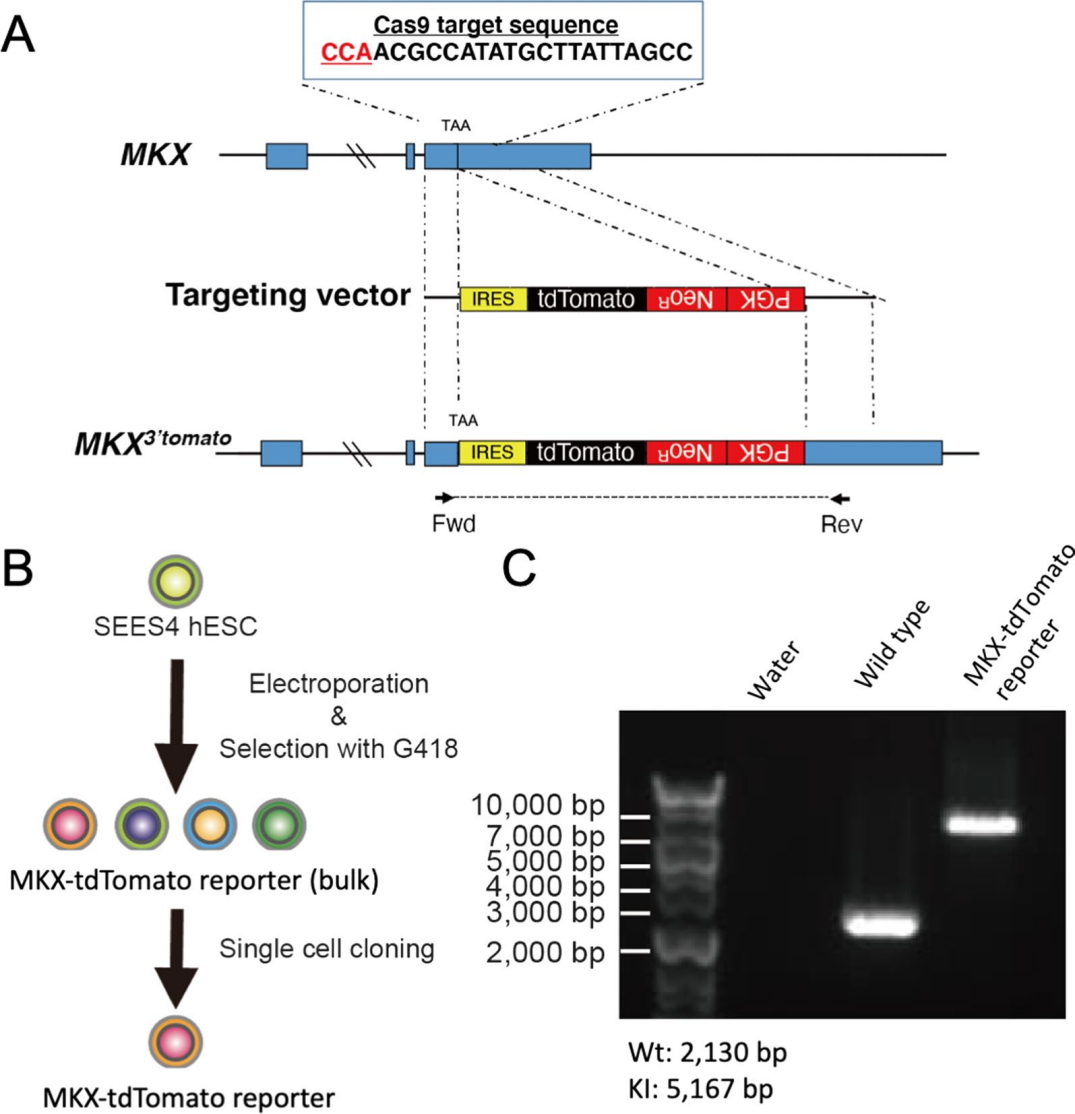
Establishment of a MKX-tdTomato reporter hPSC line. **a** The targeting cassette of the MKX-tdTomato knock-in allele. PAM sequence (CCA) is highlighted in red. **b** Generation of the MKX-tdTomato reporter hPSC line. The targeting and gRNA-Cas9 expression vector were electroporated into the hESC line SEES4. After selection with G418, single colonies were selected, expanded, and screened to identify the integration of the knock-in reporter cassette. **c**. Agarose gel electrophoresis of PCR products using forward and reverse primers that recognize sequences outside the targeting cassette. Genomic DNAs were purified from SEES4 wild type and MKX-tdTomato reporter hPSCs. WT, wild type allele; KI, knock-in allele. Full-length blot is presented in Additional file 1: Fig. S1

Figure 1A depicts the targeting strategy for the knock-in of the IRRS-tdTomato-PGK-Neo cassette at the MKX 3’ untranslated region (UTR) to achieve MKX and tdTomato coexpression. To construct the targeting vector (pEXA2J2-hMKX HA-IRES-tdTomato-PGK-Neo), primers listed in Table 2. were used and IRES-tdTomato-PGK-Neo fragments were inserted into the pEXA2J2-hMKX homology arm (pEXA2J2-hMKX HA; artificially synthesized by Eurofins) using an In-Fusion HD cloning kit (Takara).

**Table 2:** Oligos for pEXA2J2-3´MKX HA-IRES-tdTomato-PGK-Neo

Purpose	Name	Sequence
MKX 3'UTR Homology arm	F1_FW for MKX HA	CCTCAAAATGCCAACGGTACCGAGCTCGGATCC
	F1_RV for MKX HA	TAATAAGCATATGGCGGTCTAGACTCGAGGCGG
IRES-tdTomato-PGKNeo (In-fusion)	15 bp + IresTomato_PX459	GTTCCTCCTCCCTCTGGTACCGAGCTCGGATCCGCCCC
	IresTomato + 15bp_ PX459	TGGTGGTGGTATCCCGGTCTAGACTCGAGGCGGCCGC
pEXA2J2- 3'MKX HA (In-fusion)	Vec_FW for MKX HA	GCCATATGCTTATTAGCCTG
	Vec_RV for MKX HA	GTTGGCATTTTGAGGCATAGC

Guide RNAs (gRNA) were designed to target the protospacer adjacent motif (PAM) sequence-located MKX locus (CCAACGCCATATGCTTATTAGCC; the PAM sequence is indicated in bold font). The number of potential target sites in the human genome are 1 site (20 mer + PAM), 1 site (12 mer + PAM) and 389 sites (8 mer + PAM). gRNA oligos (Table 1) were designed and subcloned into the PX459 vector (Addgene, #62988) harboring a Cas9 expression cassette (PX459-MKX gRNA). To generate the MKX-tdTomato reporter line, 1 × 10^6^ SEES4 hESCs were electroporated with pEXA2J2-hMKX HA-IRES-tdTomato-PGK-Neo (1 µg) and PX459-MKX gRNA (10 µg). Selection with G418 (Life Technologies) was performed until stable colonies appeared; then, colonies were selected for expansion. To verify the precise integration of IRES-tdTomato-PGK-Neo cassette into the MKX 3’ UTR, genomic DNA was amplified using the primers in the 5’ and 3’ direction for PCR genotyping. The primers used are listed in Table 3.

**Table 3:** Genotyping primers for MKX-tdTomato reporter

Purpose	Name	Sequence
Knock-in check(WT: 2130 bp, KI: 3037 bp)	MKXreporter_OUT(F)	CCCTGACATTGTGGGAGGTC
	MKXreporter_OUT(R)	ACTGGCTGCACTATTGACCC
Cloning primers for 5′ boundary	out-F(1)	TGCAATTACAGAAACCCACCA
	ires-R(1)	CACACCGGCCTTATTCCAAG
Cloning primers for 3′ boundary	PGK-F(2)	CCAGACTGCCTTGGGAAAAG
	out-R(2)	ACCAGAGCTCAGGCTCCAAA

### Tenogenic differentiation of hPSCs

The hPSCs suspension (3 × 10^4^ cells in 1 ml of StemFit (Ajinomoto) containing 10 µM Y27632 (Wako), and 4 µl of iMatrix511 (1:250 dilution)) was seeded onto a 3.5-cm culture dish. The culture medium was replaced the next day with fresh StemFit without Y-27632. After culturing for 2 days, the cells were washed with PBS, and differentiation was induced by changing the culture medium at each time point. A chemically defined CDM2 medium was used as the basal culture medium supplemented with cytokines and chemicals to prepare each differentiation medium. The composition of the CDM2 basal medium was as follows: 50% IMDM (+ GlutaMAX; Gibco), 50% F12 (+ GlutaMAX; Gibco), 1 mg/ml polyvinyl alcohol (Sigma-Aldrich), 1% (vol/vol) chemically defined lipid concentrate (Gibco), 450 μM monothioglycerol (Sigma-Aldrich), 7 µg/ml insulin (Sigma-Aldrich), 15 μg/ml transferrin (Sigma-Aldrich), and 1% (vol/vol) penicillin–streptomycin (Gibco). On day 0, hPSCs were differentiated into the anterior primitive streak in the CDM2 medium supplemented with 30 ng/ml Activin A (R&D), 4 μM CHIR99021 (GSK3β inhibitor; Axon Medchem), 20 ng/ml FGF2 (Wako), 100 nM PIK90 (PI3K inhibitor; Millipore), and 10 μM Y-27632 for 24 h. For paraxial mesoderm (PM) differentiation, CDM2 medium containing 1 μM A-83-01 (ALK4/5/7 inhibitor; Tocris), 3 μM CHIR99021, 250 nM LDN-193189 (ALK2/3 inhibitor; ReproCELL), 20 ng/ml FGF2, and 10-μM Y-27632 was added for 24 h. On day 2, cells were cultured in a differentiation medium supplemented with 1 μM A-83-01, 250 nM LDN-193189, 1 μM C59 (PORCN inhibitor; Cellagen Technology), 500 nM PD0325901 (MEK inhibitor; Tocris), and 10 μM Y-27632 for 24 h. On day 3, somite cells were differentiated into sclerotome (SCL) cells after two days of culture in a CDM2 medium containing 5 nM SAG 21 K (SMO agonist, R&D), 1 μM C59, and 10 μM Y-27632. Subsequently, for syndetome (SYN)/tenocytes induction, cells were cultivated in the CDM2 medium supplemented with 20 ng/ml FGF8 (BioLegend) and 10 μM Y-27632 for the first three days and in the CDM2 medium supplemented with 10 ng/ml TGF β 3 (BioLegend), 10 ng/ml BMP7 (BioLegend), and 10 μM Y-27632 for the next 18 days.

### Immunocytochemistry

Cultured cells were washed with PBS, fixed with 4% paraformaldehyde (PFA) for 20 min at room temperature, and incubated with blocking solution (3% normal goat serum and 0.1% Triton X-100 in PBS) for 1 h at room temperature. Next, the cells were incubated with primary antibodies (1:200 dilution) at 4 ˚C overnight. The secondary antibodies (1:500 dilution) were subsequently added to the cells for 1 h at room temperature. After incubation, 0.1 µg/ml DAPI (Thermo Fisher) in PBS was used to counterstain the nuclei. The samples were then observed using a BZ-X710 fluorescence microscope (Keyence). The antibodies used are listed in Table 4.

**Table 4:** Antibodies used for immunocytochemistry

Name	Company	Catalog Number	Clone	Dilution
SOX2	Cell Signaling Technology	4900	L1D6A2	1:200
CDX2	Cell Signaling Technology	12306	D11D10	1:200
SOX9	MERK	AB5535		1:200
MKX	Atlas antibodies	A83377		1:200
anti-rabbit Alexa Fluor 488 Fab2	Cell Signaling Technology	4412		1:200
anti-rabbit Alexa Fluor 647 Fab2	Cell Signaling Technology	4414		1:200
anti-mouse Alexa Fluor 647 Fab2	Cell Signaling Technology	4410		1:200

### RNA extraction and quantitative reverse transcription–polymerase chain reaction

RNA was extracted using an RNeasy kit (Qiagen), and complementary DNA was synthesized using M-MLV reverse transcriptase (Thermo Fisher) and random primers (Thermo Fisher). The expression of specific genes was analyzed by qPCR using an ArialMX real-time PCR system (Agilent). The cycle parameters include denaturation at 95 °C for 30 s, annealing at 62 °C for 30 s, and elongation at 72 ˚C for 30 s. The mRNA expression levels of each gene were normalized to β -Actin (*ACTB*) and quantified using the 2^−∆∆Ct^ method. The primer sequences are listed in Table 5.

**Table 5:** qRT-PCR primers

Gene	Forward primer sequence	Reverse primer sequence
*ACTB*	AGAAAATCTGGCACCACACC	AGAGGCGTACAGGGATAGCA
*CDX2*	GGGCTCTCTGAGAGGCAGGT	CCTTTGCTCTGCGGTTCTG
*SOX9*	AAGCTCTGGAGACTTCTGAACGA	CGCCTTGAAGATGGCGTTGG
*MKX*	CTCGCAGATGACGCTAGTGC	TGGCTGTCGAACGGTATTCTT
*SCX*	GAGAAAGTTGAGCAAGGACCG	CCAGCTCAGGTCCAAGGTG
*TNMD*	TGGCCGGAGGTACCCAAAAA	AAGTAGATGCCAGTGTATCCGTTT
*COL1A1*	GACTGGTGAGACCTGCGTGT	GCCGCCATACTCGAACTGGA
*tdTomato*	CTGTTCCTGGGGCATGGCA	CGGCCATGTTGTTGTCCTCG
*ISL1*	AGATTATATCAGGTTGTACGGGATCA	ACACAGCGGAAACACTCGAT
*GATA6*	CCCACAACACAACCTACAGC	GCGAGACTGACGCCTATGTA
*OTX2*	TGCTAGAGCAGCCCTCACTC	TGGGTTTGGAGCAGTGGAACTTA

### Flow cytometry

Dissociated cells were suspended in 100 μl of 2% FBS/PBS containing 10 ng/ml DAPI. Furthermore, tdTomato expression was detected and analyzed using a CytoFLEX S flow cytometer (Beckman Coulter) and FlowJo software (FlowJo LLC), respectively.

## Results

### Establishment of an MKX-tdTomato reporter hPSC line

To visualize MKX^+^ cells at each step of tenogenic differentiation, we utilized PX459-MKX guide RNA to recombine the targeting vector harboring IRES-tdTomato-PGK-Neo cassettes to the 3’ UTR region of *MKX* in SEES4 hESCs (Fig. 1A). After electroporation of these plasmids, cells were treated with G418, and single-cell cloning was performed to establish the MKX-tdTomato reporter hPSC line (Fig. 1B). Genomic DNA was isolated from each established clone, and PCR was performed using primers that recognize the sequence of the 5’ or 3’ homology arm. As shown in Fig. 1C (Original image is Additional file 1: Fig. S1), SEES4 wild type (hereafter wild type) had wild type alleles (2130 bp), but only recombined alleles (5167 bp) were amplified in an established clone. Although off-target genome editing in possible 389 target sites (8 mer + PAM) within human genome has not been assessed in the present study, these results indicate the successful establishment of the MKX-tdTomato reporter hPSC line.

### Differentiation of SCL from hPSCs

**Fig. 2:**
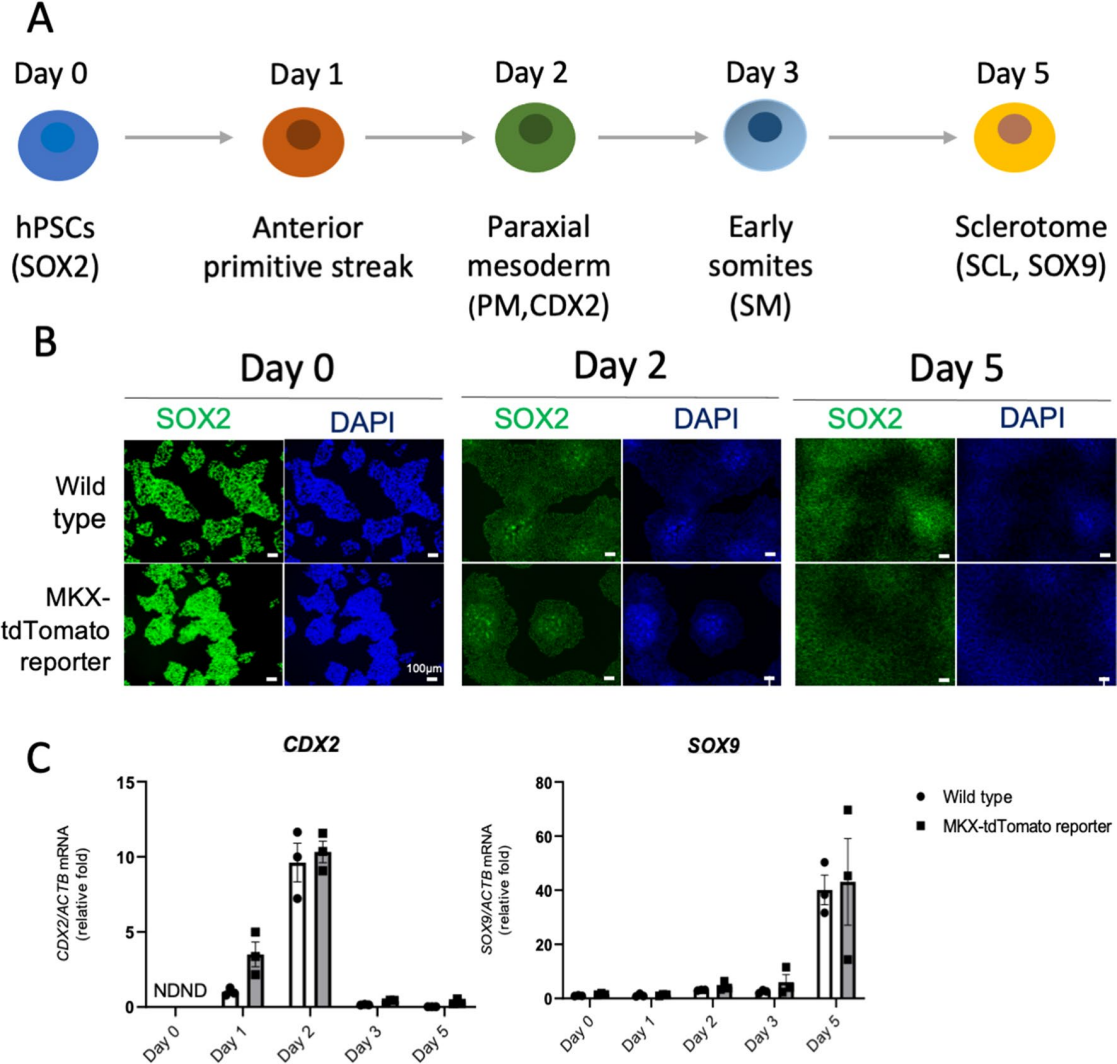
Directed differentiation of hPSCs toward PM and SCL. **a** Schematic representation of sclerotome (SCL) induction and differentiation protocol mimicking embryonic development. hPSCs were differentiated toward paraxial mesoderm (PM), somites (SM), and SCL. **b** The expression of markers for pluripotency (SOX2), PM (CDX2), and SCL (SOX9) in wild type (upper) and MKX-tdTomato reporter (lower) cells was assessed by immunocytochemistry. The nuclei were costained with DAPI. **c** qRT-PCR analysis of each marker gene on day 0, 1, 2, 3, and 5. Total RNA was extracted at each indicated time point from wild type (white column) or MKX-tdTomato reporter (gray column)-derived cells. All expression values are normalized to those of *ACTB* mRNA (n = 3, three independent experiments).

To test the differentiation capacity of our MKX-tdTomato reporter hPSCs, we performed stepwise differentiation to induce SCL that generates syndetome (SYN) or tendon progenitor cells (TPCs). During mesoderm development, pluripotent epiblast cells differentiate into the primitive streak and paraxial mesoderm (PM), subsequently producing somites. Somites are divided into two compartments, namely dermomyotome dorsally and SCL/TPCs ventrally [13, 14] (Fig. 2A). Here, we modified a previously reported protocol [13] to induce sclerotome from hPSCs. The successful transition from wild type or MKX-tdTomato reporter hPSCs to SCL was demonstrated by immunocytochemical analysis of pluripotency (SOX2, day 0), PM (CDX2, day 2), and SCL markers (SOX9, day5) (Fig. 2B). Quantitative reverse transcription–polymerase chain reaction (qRT-PCR) of the mRNA expression of each marker revealed similar results (Fig. 2C). These data suggest that MKX-tdTomato reporter hPSCs maintained the differentiation capacity to generate SCL cells.

### Induction of MKX^+^ tenocytes from SCL

**Fig. 3:**
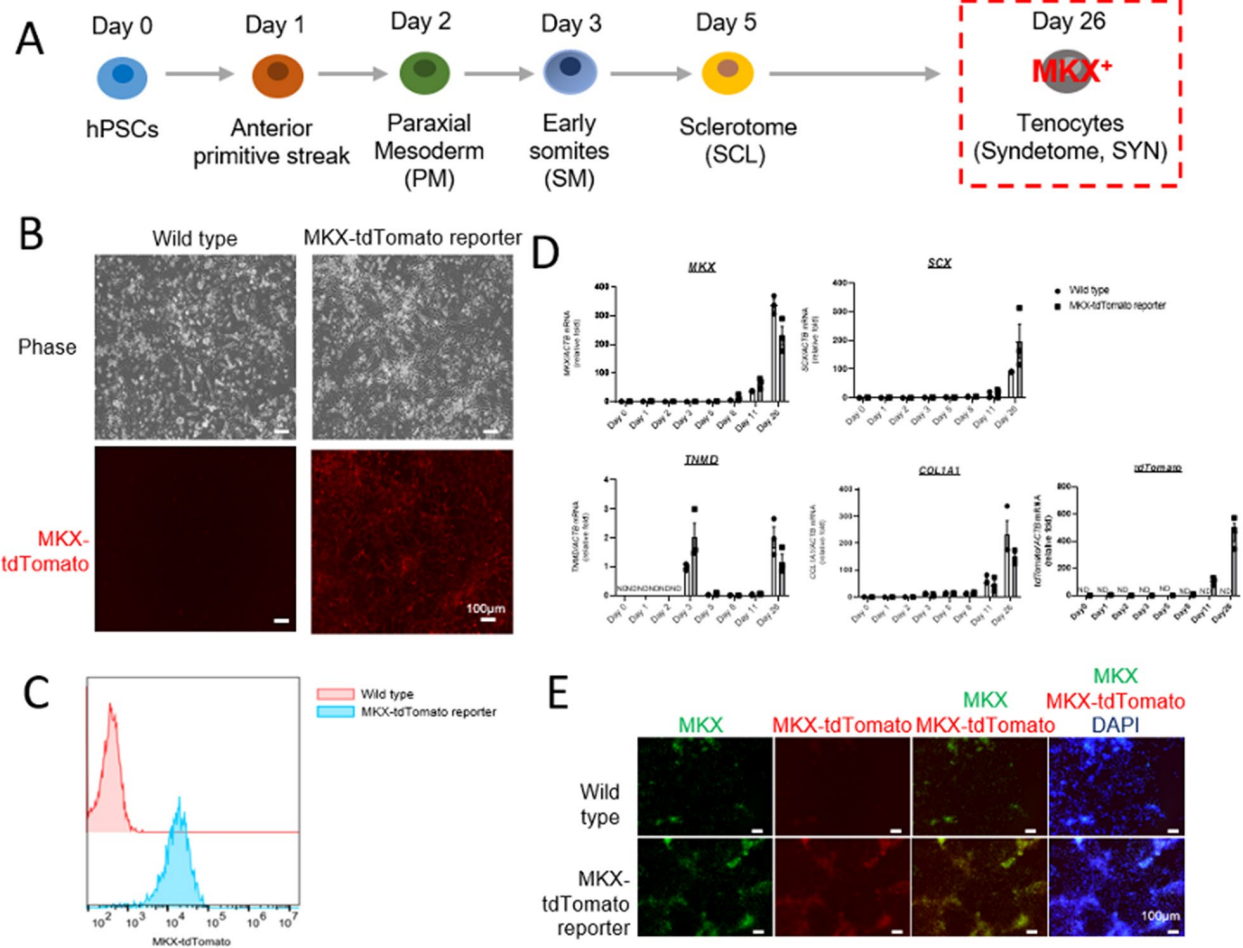
Induction of MKX^+^ tenocytes from hPSCs. **a** Schematic representation of the tenogenic differentiation protocol. hPSCs were differentiated toward PM, SM, SCL, and subsequently into tenocytes derived from syndetome (SYN), a precursor of tendon progenitor cells. **b** Morphological characteristics and MKX-tdTomato expression of wild type (upper) and MKX-tdTomato reporter (lower) cells after 26 days of tenogenic induction. **c** Flow cytometry of MKX-tdTomato expression of wild type and MKX-tdTomato reporter cells on day 26. **d** qRT-PCR analysis of tendon-specific markers. Total RNA was extracted on day 0, 1, 2, 3, 5, 8, 11, and 26 from wild type (white column) or MKX-tdTomato reporter (gray column)-derived cells. All expression values are normalized to those of *ACTB* mRNA (n = 3, three independent experiments). **e** Immunostaining of MKX in wild type (upper) and MKX-tdTomato reporter (lower)-derived cells on day 26. All MKX-tdTomato reporter cells coexpressed MKX and tdTomato

FGF8 signaling is required for SYN differentiation in the early phase, and BMP and TGFβ signaling pathways are involved in the development and maintenance of tendons and ligaments [15–17]. To induce MKX^+^ tenocytes, SCL cells were treated with FGF8/Y-27632 for three days and TGFβ3/BMP7/Y-27632 for 18 days (Fig. 3A). On day 26, MKX-tdTomato reporter-derived cells expressed tdTomato and showed spindle-shaped morphologies that resembled tenocytes (Fig. 3B). Flow cytometry revealed that almost all cells became MKX-tdTomato^+^ (Fig. 3C). As shown in Fig. 3D, the mRNA expression levels of syndetome- and tenocyte-specific marker genes (*MKX*, *SCX*, *TNMD*, and *COL1A1*) and *tdTomato* were significantly increased on day 26 after tenogenic induction. When each lineage marker expression was compared between Day 2 and Day 26, *CDX2* was downregulated at Day 26 but the expression of other lineage markers, including *OTX2* (neuroectoderm), *ISL1* (lateral plate mesoderm) and *GATA6* (definitive endoderm) was not changed (Additional file 1: Fig. S2). Importantly, the expression signature of *tdTomato* was similar to that of *MKX*, and tdTomato fluorescence was detected only at day 26 (Additional file 1: Fig. S3). Furthermore, immunocytochemical analysis showed that our protocol induced MKX and tdTomato coexpression in MKX reporter cells, confirming that the reporter system correctly visualized MKX^+^ cells (Fig. 3E). These results demonstrate that our tenogenic induction protocol generated hPSC-derived MKX-tdTomato^+^ tenocytes with high efficiency.

## Conclusions

Herein, we utilized a CRISPR/Cas9-mediated homologous recombination system to establish an hPSC-derived reporter hPSC line that allows us to visualize MKX^+^ cells by tdTomato fluorescence. Additionally, our stepwise/xeno-free induction protocol generated MKX-tdTomato^+^ tenocytes with high efficiency, which may promote further understanding of tenocyte development or provide novel insight into hPSC-based tendon regeneration.

## Supplementary Information

**Additional file 1. Supplemental Fig. S1** Full-lenght blot related to Fig. 1c.

## Data Availability

All data generated and/or analyzed during this study are included in this published article and its supplementary information.

## Abbreviations

TPCs: Tendon progenitor cells
MKX: Mohawk homeobox
hPSCs: Human pluripotent stem cells
MSCs: Mesenchymal stem cells
TSPCs: Tendon stem/progenitor cells
iPSCs: Induced pluripotent stem cells
ESCs: Embryonic stem cells
PSCs: Pluripotent stem cells
gRNA: Guide RNA
WT: Wild type
SCL: Sclerotome
SYN: Syndetome
PM: Paraxial mesoderm
SM: Somite
SOX: Sry-related HMG box
HMG: High-mobility group
TGF: Transforming growth factor
BMP: Bone morphogenetic protein
SCX: Scleraxis
TNMD: Tenomodulin
COL: Collagen
