# Supplementary material for: Establishment of a human pluripotent stem cell-derived MKX-td Tomato reporter system
Source: Stem Cell Res Ther. 2022 Nov 12;13:515. doi: 10.1186/s13287-022-03203-5 (PMC9652995; doi:10.1186/s13287-022-03203-5)
Supplement: Supplementary file 1 — Additional file 1. Supplemental Fig. S1 Full-lenght blot related to Fig. 1c. [file 13287_2022_3203_MOESM1_ESM.docx]

**
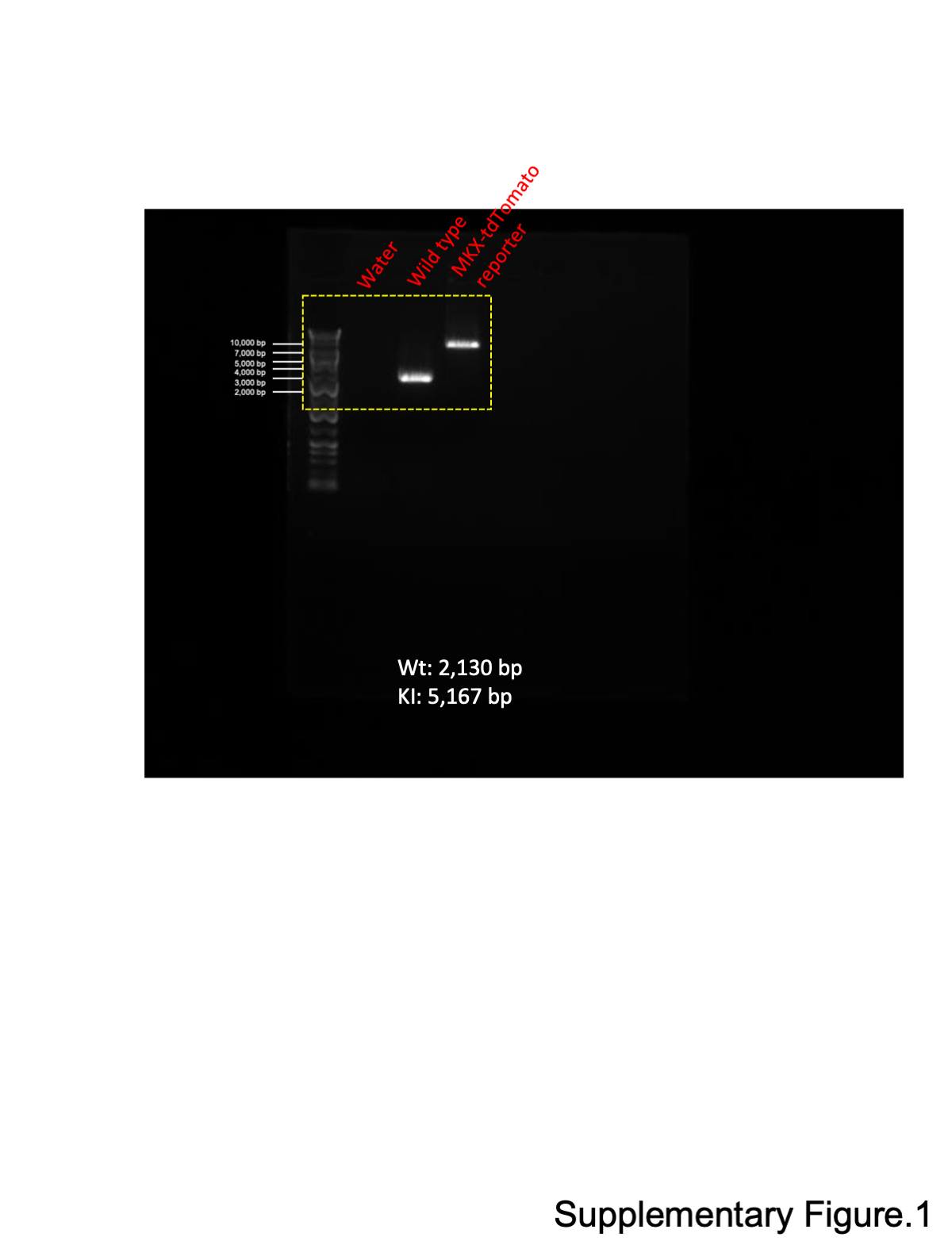
**

**Day2**

**Day26**

ND

ND

ND

ND

**Supplemental Fig. S2 Comparison of each lineage marker expression between Day 2 and Day 26**

Total RNA from wild type (white column) or MKX-tdTomato reporter (gray column)-derived cells was extracted on day 2 and 26. All expression values are normalized to those of *ACTB* mRNA (n = 3, three independent experiments).


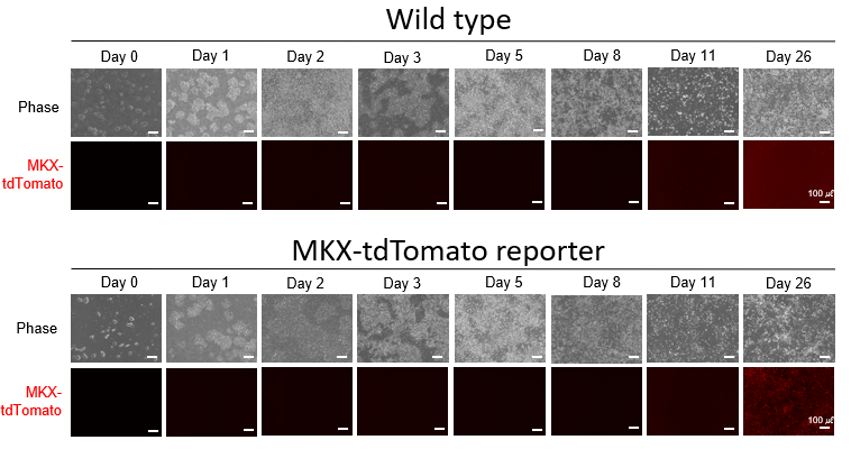
**Supplemental Fig. S3 Detection of MKX-tdTomato fluorescence during tenogenic induction**

MKX-tdTomato expression of Wild type or MKX-tdTomato reporter cells at each indicated time point during tenogenic induction was analyzed by fluorescence microscopy. tdTomato was detected only in MKX-tdTomato reporter derived cells at day 26.
